# Inverse Association Between METS-IR and Lung Cancer Risk: The Role of BMI in a Nationwide Korean Cohort

**DOI:** 10.3390/cancers17233727

**Published:** 2025-11-21

**Authors:** Bo-Kyung Shine, In Hwa Jeong, Minkook Son, Bongjo Kim, Sang Yi Moon, Jong Yoon Lee, Hye Ryeon Kim, Seok Jae Huh

**Affiliations:** 1 Department of Family Medicine, Dong-A University College of Medicine, Dong-A University Hospital, Busan 49201, Republic of Korea; sbk123@dau.ac.kr; 2 Department of Laboratory Medicine, Dong-A University College of Medicine, Dong-A University Hospital, Busan 49315, Republic of Korea; ihjeong@dau.ac.kr; 3 Department of Physiology, College of Medicine, Dong-A University, Busan 49315, Republic of Korea; physionet@dau.ac.kr (M.S.); bong15@kakao.com (B.K.); 4 Interdisciplinary Program, Department of Data Sciences Convergence, Dong-A University, Busan 49315, Republic of Korea; sang4401@dau.ac.kr; 5 Division of Gastroenterology, Department of Internal Medicine, Dong-A University College of Medicine, Busan 49315, Republic of Korea; ljyhateo@dau.ac.kr; 6 Division of Hematology and Oncology, Department of Internal Medicine, Dong-A University College of Medicine Busan 49315, Republic of Korea; hyeryeon13@dau.ac.kr

**Keywords:** lung cancer, insulin resistance, METS-IR, BMI

## Abstract

Background: Although insulin resistance has been implicated in cancer development, its specific role in lung cancer remains unclear. The metabolic score for insulin resistance (METS-IR) is a novel surrogate marker that integrates multiple metabolic parameters and has demonstrated strong predictive value for metabolic disorders. This study aimed to investigate the association between METS-IR and lung cancer incidence in a large-scale nationwide cohort. Methods: We analyzed data from 322,624 participants of the National Health Insurance Service-National Health Screening Cohort in Republic of Korea. Participants were stratified into METS-IR quartiles, and lung cancer incidence was assessed using Kaplan–Meier survival curves and Cox proportional hazards regression models. Subgroup analyses were conducted to examine the impact of body composition, particularly sarcopenia, on the association between METS-IR and lung cancer. Results: Over a median follow-up of 9.5 years, 5912 lung cancer cases were identified. Lung cancer incidence per 1000 person-years was highest in the lowest METS-IR quartile (Q1: 2.27) and decreased across quartiles (Q2: 1.93, Q3: 1.81, Q4: 1.72). In fully adjusted Cox regression models, using Q1 as the reference group, higher METS-IR quartiles were associated with a significantly lower risk of lung cancer (Q2: HR 0.91, 95% CI 0.85–0.98; Q3: HR 0.86, 95% CI 0.79–0.92; Q4: HR 0.80, 95% CI 0.74–0.86; *p* for trend < 0.001). Subgroup analyses revealed that the inverse association was more pronounced in male participants and individuals with a low body mass index. Conclusions: In this nationwide cohort study, we observed a significant inverse association between METS-IR and lung cancer risk. However, METS-IR showed limitations in fully explaining lung cancer risk based on insulin resistance alone. These findings highlight the need for future studies incorporating body composition assessments to better evaluate metabolic vulnerability.

## Simple Summary

The metabolic score for insulin resistance (METS-IR) is a simple index calculated from routine blood tests and body measurements. Although it is widely used as an indirect marker of metabolic health, METS-IR alone may not fully identify individuals who are truly at high risk. In our nationwide cohort study, people with low METS-IR values had a higher risk of developing lung cancer, particularly those with low body mass index. This suggests that some individuals may appear healthy by weight or laboratory values but still have underlying vulnerabilities such as low muscle mass or hidden visceral fat. Therefore, combining METS-IR with direct assessments of body composition—such as DEXA or BIA—may improve the ability to detect high-risk individuals and refine risk-stratification strategies. Our findings highlight the importance of evaluating metabolic health in a more comprehensive way when considering lung cancer prevention.

## 1. Background

Lung cancer is the leading cause of cancer-related mortality worldwide [1], accounting for approximately 1.79 million deaths annually [2]. Although smoking remains the most well-established risk factor, other factors such as secondhand smoke exposure, air pollution, occupational carcinogens, chronic pulmonary diseases, and genetic susceptibility have also been implicated in its pathogenesis [1]. In addition to these traditional factors, recent attention has focused on metabolic dysfunction, particularly insulin resistance, as an emerging contributor to cancer development, including lung cancer [3,4,5].

Insulin resistance has been widely implicated in the development of several cancers [6,7], including colorectal [8,9], breast [10,11], endometrial [12], and prostate cancers [13], through mechanisms involving hyperinsulinemia, chronic inflammation, and altered metabolic signaling pathways [14]. However, despite increasing attention to its role in cancer development, evidence of its association with lung cancer remains limited and inconsistent [14]. Such discrepancies underscore the importance of further investigation to clarify the role of insulin resistance in lung cancer development.

The metabolic score for insulin resistance (METS-IR) is a novel composite index developed to estimate insulin resistance using routine clinical parameters, including fasting glucose, triglycerides (TGs), body mass index (BMI), and high-density lipoprotein cholesterol (HDL-C) [15]. Unlike traditional markers such as HOMA-IR or fasting insulin levels, METS-IR does not require insulin assays and is therefore more applicable in large-scale epidemiologic studies [16,17]. By integrating multiple metabolic variables, the METS-IR offers a practical population-based approach for quantifying insulin resistance.

Given the uncertain role of insulin resistance in lung cancer and the limitations of traditional insulin resistance markers in large-scale studies, further investigations using practical and scalable measures are required. In this study, we aimed to evaluate the association between insulin resistance, as measured using the METS-IR, and the incidence of lung cancer in a nationally representative Korean cohort.

## 2. Methods

### 2.1. Data Source and Study Population

Approximately 97% of the Republic of Korea population benefits from the mandatory health insurance system established by the government, with the remaining 3% covered by the Medical Aid Program. In Republic of Korea, a biennial health examination initiative ensures extensive coverage for all employees, regardless of age, as well as individuals aged 40 and above.

The National Health Insurance Service-National Health Screening Cohort (NHIS-HealS) is a population-based database constructed from the national health screening records and medical claims data in Republic of Korea. In this study, we included individuals who underwent a national health screening program between 2010 and 2011. The NHIS-HealS database provides comprehensive information on demographics, diagnoses, procedures, prescriptions, and lifestyle factors, making it suitable for large-scale epidemiological studies [18,19]. For this study, we used the NHIS-HealS database, which encompasses comprehensive medical claims information, including prescription records, procedures, surgeries, insurance premium payments, and other details related to both inpatient and outpatient healthcare services. In addition, the database includes health screening data such as laboratory tests, physical measurements, and self-reported lifestyle questionnaires.

**Figure 1:**
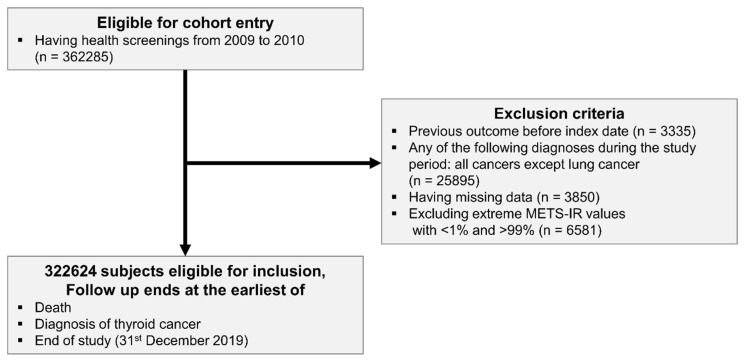
The flow of study population.

Participants (*n* = 364,757) who underwent a health screening program between 2010 and 2011 were initially identified. The participants were diagnosed and categorized using the International Classification of Diseases, version 10 (ICD-10). To refine the cohort, we excluded individuals with a previous diagnosis of lung cancer (*n* = 3335), those diagnosed with cancers other than lung cancer during the observation period (*n* = 25,895), and those with missing data (*n* = 3850). Participants with missing values for fasting glucose, triglyceride, HDL-cholesterol, or covariates were excluded from the analysis (complete-case analysis). No imputation was performed. We excluded 6581 participants with METS-IR values below the 1st percentile or above the 99th percentile. The final cohort comprised 322,624 participants. The follow-up period continued until 31 December 2019, or until death or a diagnosis of lung cancer was made (Figure 1).

The Institutional Review Board of Dong-A University College of Medicine exempted this study protocol from review because of its retrospective design. The investigators used only de-identified open clinical data for analytical purposes (DAUHIRB-EXP-25-025).

### 2.2. Definitions of Surrogate Markers for Insulin Resistance: METS-IR

METS-IR was calculated using the following formula: ln [(2 × fasting blood glucose (FBG) in mg/dL + serum TG level in mg/dL) × BMI in kg/m^2^]/ln [HDL-C level in mg/dL] [15]. At baseline, 322,624 participants were divided into four quartiles (Q1, Q2, Q3, and Q4) based on their METS-IR results, and the outcome variables were identified during follow-up.

### 2.3. Study Outcomes: Lung Cancer

The primary outcome of the study was the incidence of lung cancer, which was identified using the ICD-10 codes C33 and C34. Previous research has demonstrated that using ICD-10 codes from the National Health Insurance Service database to diagnose lung cancer has a high sensitivity (95.0%; 95% confidence interval [CI], 94.8%–95.1%) and positive predictive value (88.9%; 95% CI, 88.8%–89.1%) [20].

### 2.4. Covariates

Information on demographic and clinical characteristics—including age, sex, and major comorbidities such as hypertension, diabetes, and dyslipidemia—was obtained from the linked health screening and claims databases. Additional details are summarized in Supplementary Table S1. Household income was categorized into quartiles, and place of residence was grouped into urban or rural areas. The Charlson Comorbidity Index (CCI) was calculated based on relevant diagnostic codes to reflect the overall burden of chronic illness [21]. Laboratory data, including hemoglobin concentration and estimated glomerular filtration rate (eGFR), were also incorporated. Lifestyle information—including cigarette smoking, alcohol intake, and engagement in regular physical activity—was assessed through standardized self-reported questionnaires.

### 2.5. Statistical Analyses

Categorical variables were summarized as counts and percentages, whereas continuous variables were reported as means with standard deviations. Kaplan–Meier curves were generated to compare cumulative lung cancer incidence across METS-IR quartiles. Cox proportional hazards regression was used to estimate hazard ratios (HRs) and 95% confidence intervals (CIs) for the association between METS-IR and lung cancer. The proportional hazards assumption was evaluated using Schoenfeld residuals, which indicated no violation of model assumptions. Multivariable models were adjusted for age, sex, income level, residential area, hypertension, diabetes, dyslipidemia, CCI, hemoglobin, eGFR, smoking, alcohol consumption, and physical activity. A restricted cubic spline model was applied to explore potential nonlinear relationships between METS-IR [22]. and lung cancer risk, with knots placed at the 5th, 35th, 65th, and 95th percentiles of METS-IR values and the mean serving as the reference.

All analyses were performed using SAS version 9.4 (SAS Institute Inc., Cary, NC, USA) and R version 3.6.0 (R Foundation for Statistical Computing, Vienna, Austria). Statistical significance was defined as a two-sided *p*-value < 0.05.

## 3. Results

### 3.1. Baseline Characteristics

A total of 322,624 participants were enrolled in this study and categorized into four groups using the METS-IR. The corresponding METS-IR values for each quartile (Q1, Q2, Q3, and Q4) were 28.6 ± 2.0, 33.2 ± 1.3, 36.9 ± 1.4, and 42.7 ± 3.0, respectively. Table 1 shows the varied results, such as sex, age, income level, residence, underlying disease, BMI, blood pressure, FBG, total cholesterol, TG, HDL-C, low-density lipoprotein cholesterol, hemoglobin, GFR, smoking, alcohol consumption, and regular exercise, for the quartiles. Significant differences (*p* < 0.001) were observed among the four groups regarding the characteristics during the 2010–2011 health screening. Additional table files show the baseline characteristics of each sex in detail [see Supplementary Tables S2 and S3].

### 3.2. Incidence of Lung Cancer According to METS-IR

**Figure 2:**
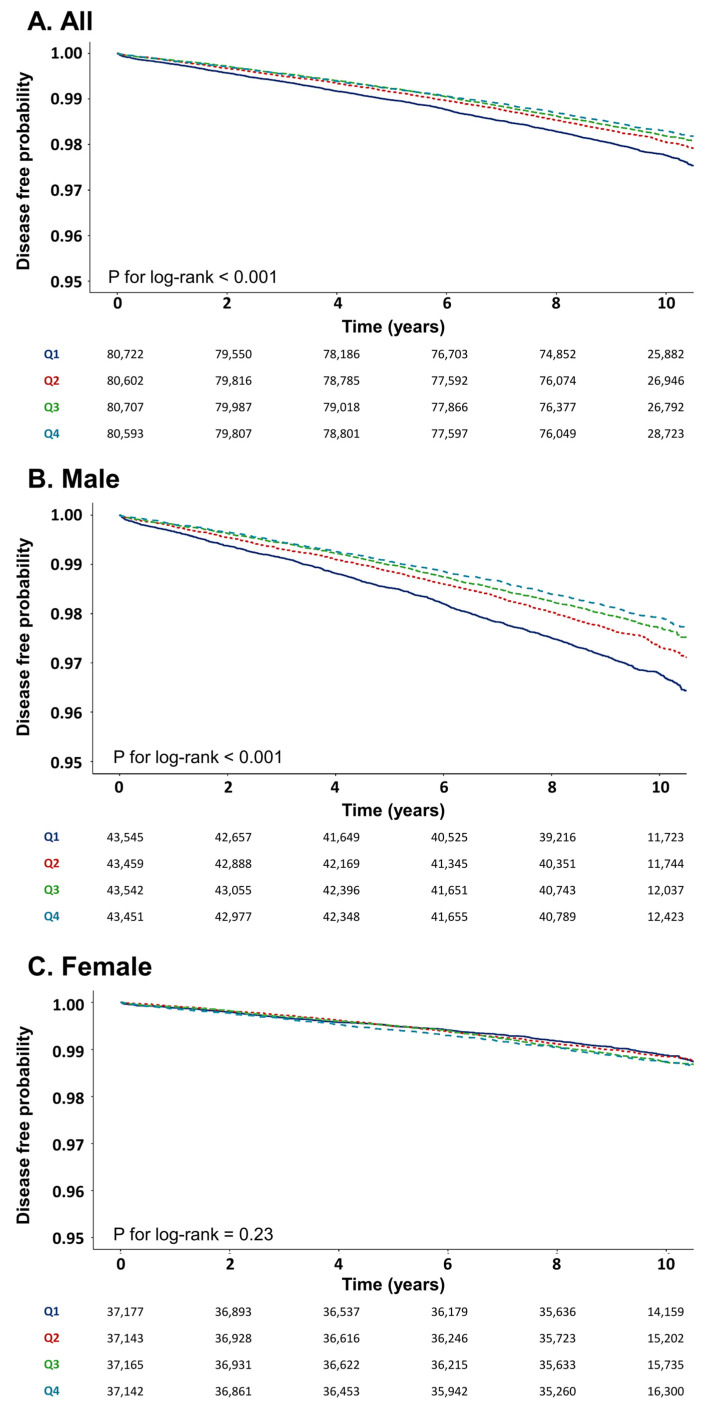
Kaplan–Meier curve for association between METS-IR and lung cancer.

The study population was followed up for a median of 9.5 years, during which 5912 lung cancer cases occurred. During the observation period, we conducted Kaplan–Meier analysis to compare the cumulative incidence rates of lung cancer among the four quartiles of the METS-IR. The results indicated a sequential decrease in the incidence of lung cancer from Q1 to Q4 (*p* < 0.001, log-rank test) (Figure 2).

Table 2 shows the crude and adjusted HRs with 95% CIs for the association between lung cancer incidence rate and the METS-IR quartiles. In the crude analysis, the incidence rates of lung cancer for the METS-IR quartiles were as follows: 2.27, 1.93, 1.81, and 1.72 cases per 1000 person-years for Q1, Q2, Q3, and Q4, respectively. The corresponding HRs and 95% CIs for lung cancer incidence rates were 1 (reference) for Q1, 0.85 (95% CI, 0.79–0.91) for Q2, 0.79 (95% CI, 0.74–0.85) for Q3, and 0.75 (95% CI, 0.70–0.81) for Q4, in the same groups. After adjustment for variables, the corresponding HRs and 95% CIs for lung cancer incidence rates were 1 (reference) for Q1, 0.91 (95% CI, 0.85–0.98) for Q2, 0.86 (95% CI, 0.79–0.92) for Q3, and 0.80 (95% CI, 0.74–0.86) for Q4. Both crude and adjusted analyses showed a statistically significant decrease in the incidence of lung cancer from Q1 to Q4 in the METS-IR.

**Figure 3:**
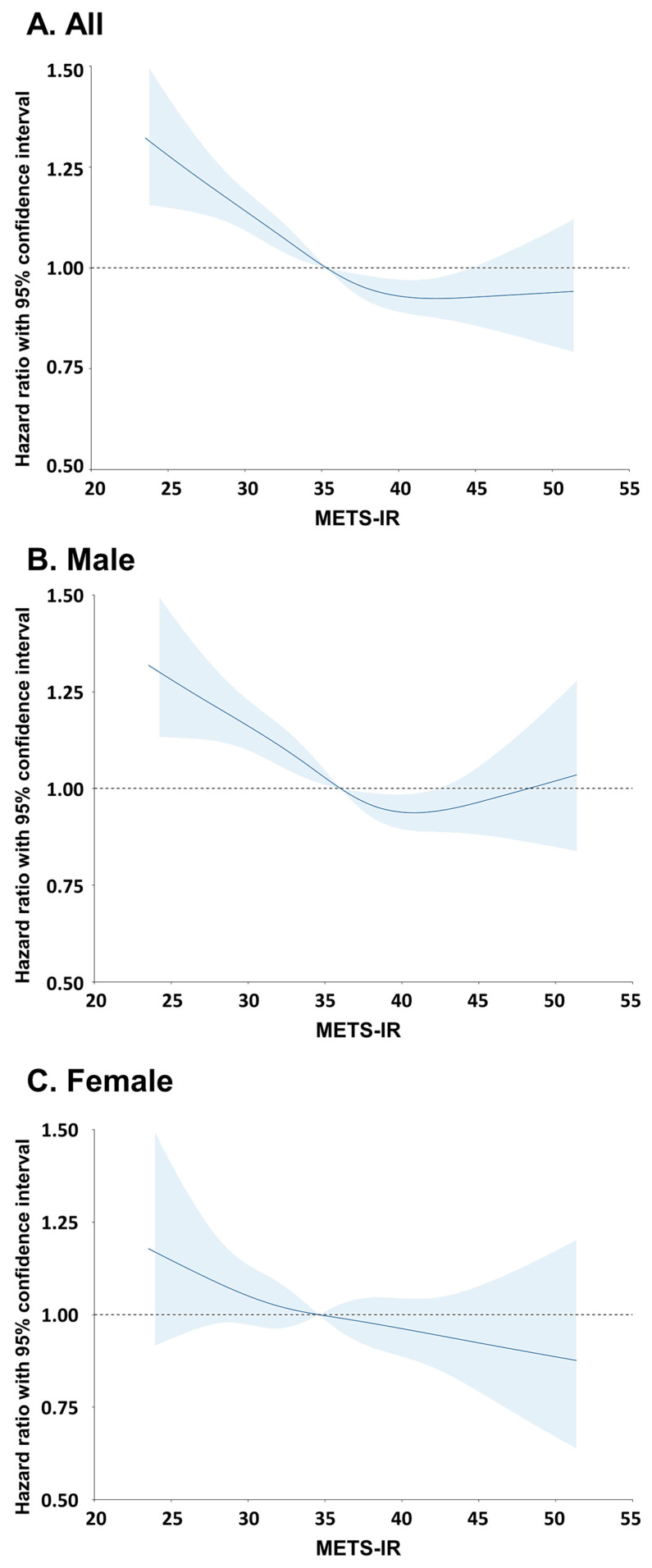
Restricted cubic spline of hazard ratio with 95% confidence intervals for lung cancer according to METS-IR.

Figure 3 shows a restricted cubic spline for the correlation between METS-IR and lung cancer incidence. The nonlinear relationship between METS-IR and the incidence of lung cancer was evaluated by a restricted cubic spline using a multivariable adjusted model to depict the trend of HR with respect to METS-IR. The restricted cubic spline curves showed that the incidence of lung cancer decreased with increasing METS-IR for all participants.

### 3.3. Subgroup Analysis

**Figure 4:**
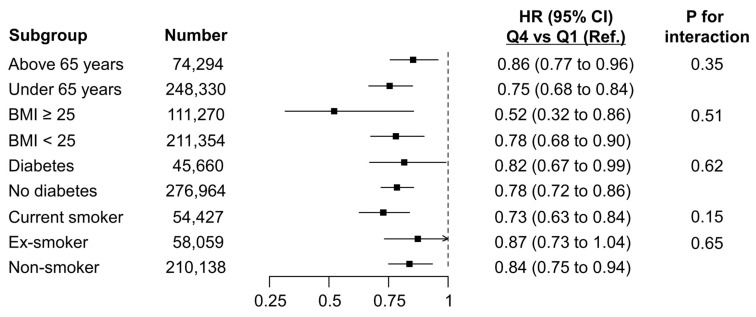
Subgroup analyses according to METS-IR.

Figure 4 shows HR comparisons from Q4 to Q1 of the METS-IR within subgroups categorized by age (<65 years and ≥65 years), obesity (defined as BMI ≥ 25), diabetes, and smoking status (current smoker, ex-smoker, and non-smoker). According to the subgroup analysis, all examined subgroups showed a decrease in HR at Q4 compared to that at Q1 of the METS-IR, consistent with the overall population. Notably, this decrease was most pronounced in the obesity subgroup.

## 4. Discussion

In this nationwide cohort of 322,624 participants, 5912 incident lung cancer cases were identified over a median follow-up of 9.5 years. Participants in the highest METS-IR quartile (Q4) showed a significantly lower risk of lung cancer than those in the lowest quartile (Q1), with an adjusted HR of 0.80 (95% CI: 0.74–0.86). Subgroup analysis revealed that the inverse relationship between METS-IR and lung cancer risk was consistently observed across all subgroups and was particularly pronounced in participants with obesity. This quartile-based categorization of METS-IR follows a widely used analytical structure in previous epidemiologic research, where METS-IR quartiles were employed to assess metabolic risk gradients and disease incidence [23,24].

Recent studies have highlighted the complex relationship between changes in body weight and the risk of lung cancer. A U-shaped association has been reported, with both major weight loss and gain linked to increased risk, even among never-smokers [25]. Similarly, a prospective analysis of the PLCO Trial found that an increase in BMI over time was associated with a lower risk of non-small cell lung cancer [26]. These findings suggest that BMI fluctuations may reflect deeper metabolic disturbances; however, BMI alone does not distinguish between fat and muscle mass or account for metabolic quality [27,28]. Supporting this, a recent meta-analysis of 31 studies reported that while insulin resistance and diabetes mellitus were positively associated with lung cancer risk, a high BMI was inversely associated with lung cancer risk [3]. These findings further underscore that BMI alone may inadequately reflect underlying metabolic vulnerability. Moreover, weight fluctuation itself has been associated with an increased risk of cardiovascular disease and mortality, suggesting that dynamic changes in body weight may reflect underlying metabolic instability [29]. In addition, normal-weight obesity, characterized by excess fat mass despite a normal BMI, has been associated with increased cardiometabolic risk [30]. These hidden forms of adiposity highlight that BMI alone is inadequate to fully capture the complex metabolic disturbances underlying lung cancer risk.

Supporting this finding, a Japanese study using bioelectrical impedance analysis found that the fat-to-muscle mass ratio, rather than either component alone, was independently associated with insulin resistance [31], underscoring the importance of body composition quality in assessing metabolic vulnerability. A comparative study of lean and obese individuals with metabolic syndrome showed similar levels of insulin resistance across both groups [32], underscoring that body size alone may not accurately capture metabolic vulnerability—an insight consistent with our findings regarding low METS-IR and lung cancer risk. The more pronounced inverse association observed in males may reflect sex-related differences in fat distribution, hormonal balance, and behavioral factors such as smoking or alcohol consumption [33]. Men generally exhibit greater visceral fat accumulation and a higher prevalence of smoking, both of which may influence metabolic profiles and inflammatory responses differently compared with women [34]. These differences could partially explain the stronger association between lower METS-IR and lung cancer risk observed in men.

Although METS-IR is widely used as a practical surrogate of insulin resistance in epidemiological studies, it may not fully capture the heterogeneity of metabolic health in lean populations. Individuals with low BMI or METS-IR values may still exhibit metabolic vulnerability due to underlying factors such as mitochondrial dysfunction [35,36], systemic inflammation [37], or inadequate nutrient sensing [38]. In this context, “metabolically unhealthy lean” (MUL) refers to individuals who have a normal BMI but display metabolically adverse characteristics—including insulin resistance, visceral adiposity, sarcopenia, or chronic inflammation—despite their lean appearance. This notion of “metabolically unhealthy lean” phenotypes is supported by previous studies linking low adiposity and poor metabolic profiles with increased morbidity and mortality [39,40]. Therefore, when interpreting the inverse association between METS-IR and lung cancer, it is important to consider that low scores may, in some cases, reflect underlying frailty or nontraditional forms of metabolic dysfunction rather than a truly healthy metabolic state.

This study had several strengths, including the use of a large-scale nationally representative dataset, long-term follow-up, and the novel application of METS-IR as a metabolic marker.

METS-IR has previously demonstrated predictive value in various contexts, including all-cause and cardiovascular mortality in the general US population using National Health and Nutrition Examination Survey data [41], as well as mortality among older adults in Republic of Korea [42]. Moreover, it has been associated with the development of non-alcoholic fatty liver disease [43], chronic kidney disease [44], and polycystic ovary syndrome [45]. Given its simplicity and reproducibility, METS-IR can be easily applied in both research and clinical practice as a marker of metabolic vulnerability. Because it relies solely on routine laboratory and anthropometric parameters, it provides a cost-effective approach for population-based risk stratification and early identification of individuals at higher metabolic or oncologic risk. However, METS-IR alone may not be sufficient to fully capture metabolic heterogeneity. Combining METS-IR with measures of body composition—such as DEXA or BIA—is essential for enhancing the precision of lung cancer risk prediction and for developing more personalized preventive strategies.

This study also has several limitations. First, direct measurements of muscle mass were not available, which limited our ability to confirm the role of sarcopenia in our findings. Future studies incorporating direct assessments of sarcopenia, such as dual-energy X-ray absorptiometry (DEXA) or bioelectrical impedance analysis (BIA), are necessary to validate these observations. Second, as this was an observational study, we could not establish causality between METS-IR and lung cancer risk. Although we adjusted for multiple confounders, residual confounding from unmeasured variables such as nutritional status and inflammatory markers may still be present. These findings suggest that METS-IR may serve as an associated indicator for cancer risk stratification. Future studies should integrate additional metabolic biomarkers and direct muscle mass assessments to provide a more comprehensive understanding of this association.

From a clinical standpoint, our findings highlight the need to incorporate both metabolic indices and body composition assessments into lung cancer risk-prediction models. Traditional screening frameworks that focus on BMI or insulin resistance may overlook high-risk individuals with low muscle mass or altered metabolic profiles. Therefore, integrating METS-IR with methods such as DEXA or BIA can improve early detection and prevention strategies.

## 5. Conclusions

This study provides novel insights into the association between insulin resistance and the risk of lung cancer in a nationwide population. An inverse relationship between METS-IR and lung cancer was consistently observed across subgroups and was particularly pronounced in older adults and individuals with obesity. These findings indicate that low METS-IR is associated with, but may not directly determine, lung cancer risk, particularly among lean individuals who may harbor hidden metabolic vulnerabilities. To improve cancer risk prediction, both stratified analyses and body composition assessments should be routinely incorporated into standard metabolic evaluations as part of regular health screenings. Furthermore, future research should consider body composition alongside insulin resistance when evaluating cancer risk to better understand the potential contribution of metabolic dysfunction to cancer development and guide more precise preventive strategies.

## Figures and Tables

**Table 1:** Baseline characteristics of study population according to METS-IR.

All Subjects (N = 322,624)	METS-IR				*p*-Value
	1st Quartile, Q1 (N = 80,722)	2nd Quartile, Q2 (N = 80,602)	3rd Quartile, Q3 (N = 80,707)	4th Quartile, Q4 (N = 80,593)	
**Demographics**					
Age (years)	58.4 (9.1)	58.5 (8.6)	58.9 (8.5)	59.2 (8.6)	<0.001
Sex (%)					1.00
Male	43,545 (53.9)	43,459 (53.9)	43,542 (54.0)	43,451 (53.9)	
Female	37,177 (46.1)	37,143 (46.1)	37,165 (46.0)	37,142 (46.1)	
Income Level (%)					<0.001
1st quartile	11,572 (14.3)	11,184 (13.9)	11,105 (13.8)	11,295 (14.0)	
2nd quartile	17,814 (22.1)	16,675 (20.7)	16,069 (19.9)	16,062 (19.9)	
3rd quartile	23,198 (28.7)	23,374 (29.0)	23,914 (29.6)	24,670 (30.6)	
4th quartile	28,138 (34.9)	29,369 (36.4)	29,619 (36.7)	28,566 (35.4)	
Residence (%)					<0.001
Urban	53,251 (66.0)	52,349 (64.9)	51,938 (64.4)	50,273 (62.4)	
Rural	27,471 (34.0)	28,253 (35.1)	28,769 (35.6)	30,320 (37.6)	
**Underlying disease**					
Hypertension (%)	26,472 (32.8)	34,026 (42.2)	40,506 (50.2)	49,054 (60.9)	<0.001
Diabetes (%)	5112 (6.3)	8514 (10.6)	12,498 (15.5)	19,536 (24.2)	<0.001
Dyslipidemia (%)	18,676 (23.1)	26,325 (32.7)	34,599 (42.9)	48,544 (60.2)	<0.001
Charlson Comorbidity Index					<0.001
0	42,623 (52.8)	38,925 (48.3)	36,075 (44.7)	31,887 (39.6)	
1	21,672 (26.8)	22,307 (27.7)	22,424 (27.8)	21,939 (27.2)	
2	9481 (11.7)	10,540 (13.1)	11,429 (14.2)	12,523 (15.5)	
≥3	6946 (8.6)	8830 (11.0)	10,779 (13.4)	14,244 (17.7)	
**Health Screening**					
Body Mass Index (kg/m^2^)	21.1 (1.6)	23.2 (1.4)	24.8 (1.5)	27.0 (2.1)	<0.001
Systolic Blood Pressure (mmHg)	121.5 (15.3)	124.3 (15.0)	126.4 (14.8)	128.8 (14.8)	<0.001
Diastolic Blood Pressure (mmHg)	75.4 (9.9)	77.0 (9.8)	78.2 (9.8)	79.7 (9.8)	<0.001
Fasting Blood Glucose (mg/dL)	94.1 (16.5)	98.1 (20.2)	102.0 (23.7)	109.3 (32.3)	<0.001
Total Cholesterol (mg/dL)	197.7 (35.3)	200.5 (36.8)	201.6 (38.0)	201.6 (39.0)	<0.001
Triglyceride (mg/dL)	93.0 (48.7)	117.4 (56.0)	144.3 (70.7)	193.4 (103.2)	<0.001
HDL Cholesterol (mg/dL)	64.7 (27.3)	55.8 (12.0)	50.9 (10.8)	45.1 (9.7)	<0.001
LDL Cholesterol (mg/dL)	115.8 (35.6)	121.2 (35.9)	122.0 (37.6)	118.2 (39.3)	<0.001
Hemoglobin (g/dL)	13.6 (1.4)	13.8 (1.5)	13.9 (1.5)	14.1 (1.5)	<0.001
Glomerular Filtration Rate (mL/min/1.73 m^2^)	80.4 (30.3)	79.1 (30.3)	77.8 (30.9)	76.9 (32.4)	<0.001
Current Smoker (%)	15,015 (18.6)	12,925 (16.0)	12,877 (16.0)	13,610 (16.9)	<0.001
Alcohol Drink (%)	32,892 (40.7)	32,877 (40.8)	32,220 (39.9)	30,843 (38.3)	<0.001
Regular Exercise (%)	3909 (4.8)	3948 (4.9)	3724 (4.6)	3418 (4.2)	<0.001
METS-IR	28.6 (2.0)	33.2 (1.3)	36.9 (1.4)	42.7 (3.0)	<0.001

**Table 2:** Hazard ratio and 95% confidence interval for incidence of lung cancer according to METS-IR.

Subjects (N = 322,624)	Events	Follow-up Duration (Person-Years)	Incidence Rate (per 1000 Person-Years)	Hazard Ratio (95% Confidence Intervals)			
				Crude	*p*-Value	Adjusted *	*p*-Value
Total							
Q1 (N = 80,722)	1722	758,787	2.27	1.00 (reference)		1.00 (reference)	
Q2 (N = 80,602)	1480	765,719	1.93	0.85 (0.79–0.91)	<0.001	0.91 (0.85–0.98)	0.009
Q3 (N = 80,707)	1390	766,717	1.81	0.79 (0.74–0.85)	<0.001	0.86 (0.79–0.92)	<0.001
Q4 (N = 80,593)	1320	765,634	1.72	0.75 (0.70–0.81)	<0.001	0.80 (0.74–0.86)	<0.001
Male							
Q1 (N = 43,545)	1318	400,614	3.29	1.00 (reference)		1.00 (reference)	
Q2 (N = 43,459)	1068	404,169	2.64	0.80 (0.74–0.87)	<0.001	0.92 (0.85–0.99)	0.04
Q3 (N = 43,542)	938	409,295	2.29	0.70 (0.64–0.76)	<0.001	0.85 (0.78–0.93)	<0.001
Q4 (N = 43,451)	861	408,439	2.11	0.64 (0.59–0.70)	<0.001	0.80 (0.73–0.88)	<0.001
Female							
Q1 (N = 37,177)	404	356,899	1.13	1.00 (reference)		1.00 (reference)	
Q2 (N = 37,143)	412	360,287	1.14	1.01 (0.88–1.16)	0.87	0.94 (0.82–1.07)	0.35
Q3 (N = 37,165)	452	360,501	1.25	1.11 (0.97–1.27)	0.14	0.94 (0.82–1.08)	0.36
Q4 (N = 37,142)	459	360,277	1.27	1.13 (0.98–1.29)	0.08	0.88 (0.76–1.01)	0.07

* The model was adjusted for age, sex, income level, residence, hypertension, diabetes, dyslipidemia, Charlson comorbidity index, hemoglobin level, glomerular filtration rate, smoking, alcohol drink, and regular exercise status.

## Data Availability

The datasets generated and/or analyzed in the current study are available from the corresponding author upon reasonable request. Requests should be directed to BKS.

## Supplementary Materials

The following supporting information can be downloaded at: https://www.mdpi.com/article/10.3390/cancers17233727/s1, Supplementary Table S1. Definitions for clinical variables. Supplementary Table S2. Baseline characteristics of study population according to METS-IR (male). Supplementary Table S3. Baseline characteristics of study population according to METS-IR (female).

## Abbreviations

METS-IR	Metabolic score for insulin resistance
BMI	Body mass index
HDL-C	High-density lipoprotein cholesterol
TG	Triglyceride
NHIS-HealS	National Health Insurance Service-National Health Screening Cohort
ICD-10	International Classification of Diseases, version 10
FBG	Fasting blood glucose
CI	Confidence interval
CCI	Charlson comorbidity index
GFR	Glomerular filtration rate
HR	Hazard ratio
BIA	Bioelectrical impedance analysis
DEXA	Dual-energy X-ray absorptiometry
