# Supplementary material for: Inverse Association Between METS-IR and Lung Cancer Risk: The Role of BMI in a Nationwide Korean Cohort
Source: Cancers (Basel). 2025 Nov 21;17(23):3727. doi: 10.3390/cancers17233727 (PMC12691392; doi:10.3390/cancers17233727)
Supplement: Supplementary file 1 [file cancers-17-03727-s001.zip › Supplementary Table S1.pdf]

Supplementary Table S1. Definitions for clinical variables.

Clinical variables	ICD-10 codes + Claim codes	Health screening
Hypertension	I10-I11 + Prescription of anti-hypertensive drugs ≥ 1	Systolic blood pressure ≥ 140 mmHg or Diastolic blood pressure ≥ 90 mmHg
Diabetes	E10-E14 + Prescription of anti-diabetic drugs ≥ 1	Fasting blood glucose ≥ 126 mg/dL
Dyslipidemia	E78 + Prescription of lipid-lowering drugs ≥ 1	Total cholesterol 240 mg/dL or<br>LDL cholesterol 190 mg/dL or<br>Triglyceride 500 mg/dL or<br>HDL cholesterol < 40 mg/dL
Lung cancer	C33, C34	
