# Supplementary material for: Inverse Association Between METS-IR and Lung Cancer Risk: The Role of BMI in a Nationwide Korean Cohort
Source: Cancers (Basel). 2025 Nov 21;17(23):3727. doi: 10.3390/cancers17233727 (PMC12691392; doi:10.3390/cancers17233727)
Supplement: Supplementary file 1 [file cancers-17-03727-s001.zip › Supplementary Table S3.pdf]

Supplementary Table S3. Baseline characteristics of study population according to METS-IR (female).

All subjects (N=148627)	METS-IR (female)				P-value
	1st quartile, Q1 (N=37177)	2nd quartile, Q2 (N=37143)	3rd quartile, Q3 (N=37165)	4th quartile, Q4 (N=37142)	
**Demographics**					
Age (years)	57.4 (8.9)	58.7 (8.7)	60.2 (8.8)	61.4 (8.8)	< 0.001
Income level (%)					< 0.001
1st quartile	6396 (17.2)	6451 (17.4)	6431 (17.3)	6413 (17.3)	
2nd quartile	8778 (23.6)	8673 (23.4)	8524 (22.9)	8609 (23.2)	
3rd quartile	9927 (26.7)	10622 (28.6)	11179 (30.1)	11662 (31.4)	
4th quartile	12076 (32.5)	11397 (30.7)	11031 (29.7)	10458 (28.2)	
Residence (%)					< 0.001
Urban	24665 (66.3)	23591 (63.5)	23126 (62.2)	22147 (59.6)	
Rural	12512 (33.7)	13552 (36.5)	14039 (37.8)	14995 (40.4)	
**Underlying disease**					
Hypertension (%)	10894 (29.3)	14767 (39.8)	18625 (50.1)	23617 (63.6)	< 0.001
Diabetes (%)	1636 (4.4)	2866 (7.7)	4628 (12.5)	8141 (21.9)	< 0.001
Dyslipidemia (%)	9984 (26.9)	13056 (35.2)	16587 (44.6)	22004 (59.2)	< 0.001
Charlson comorbidity index					< 0.001
0	18943 (51.0)	16654 (44.8)	14812 (39.9)	12350 (33.3)	
1	10434 (28.1)	10841 (29.2)	10844 (29.2)	10520 (28.3)	
2	4610 (12.4)	5336 (14.4)	5907 (15.9)	6432 (17.3)	

≥3	3190 (8.6)	4312 (11.6)	5602 (15.1)	7840 (21.1)	
**Health screening**					
Body mass index (kg/m^2)	20.9 (1.5)	23.0 (1.4)	24.7 (1.6)	27.2 (2.3)	< 0.001
Systolic blood pressure (mmHg)	119.1 (15.1)	122.5 (15.2)	125.4 (15.3)	128.5 (15.3)	< 0.001
Diastolic blood pressure (mmHg)	73.7 (9.8)	75.4 (9.7)	76.9 (9.8)	78.5 (9.8)	< 0.001
Fasting blood glucose (mg/dL)	91.9 (13.9)	95.2 (17.0)	99.0 (20.8)	106.1 (29.5)	< 0.001
Total cholesterol (mg/dL)	204.1 (35.8)	205.6 (37.2)	206.9 (38.8)	206.9 (39.8)	< 0.001
Triglyceride (mg/dL)	88.3 (44.4)	108.9 (48.8)	133.2 (63.0)	173.9 (89.6)	< 0.001
HDL cholesterol (mg/dL)	67.2 (29.7)	58.1 (12.1)	53.4 (11.8)	47.6 (10.3)	< 0.001
LDL cholesterol (mg/dL)	120.7 (35.0)	125.5 (35.3)	126.9 (37.8)	124.4 (38.2)	< 0.001
Hemoglobin (g/dL)	12.7 (1.1)	12.8 (1.1)	12.9 (1.1)	13.0 (1.1)	< 0.001
Glomerular filtration rate (mL/min/1.73 m^2)	79.4 (25.3)	79.0 (27.8)	77.5 (25.5)	76.5 (26.1)	< 0.001
Current smoker (%)	639 (1.7)	538 (1.4)	537 (1.4)	601 (1.6)	0.01
Alcohol drink (%)	5946 (16.0)	5363 (14.4)	4865 (13.1)	4035 (10.9)	< 0.001
Regular exercise (%)	1520 (4.1)	1442 (3.9)	1463 (3.9)	1277 (3.4)	< 0.001
METS-IR	28.1 (1.8)	32.3 (1.0)	36.0 (1.1)	41.9 (3.1)	< 0.001
